# Genetic variability of the P120' surface protein gene of *Mycoplasma hominis *isolates recovered from Tunisian patients with uro-genital and infertility disorders

**DOI:** 10.1186/1471-2334-7-142

**Published:** 2007-12-05

**Authors:** Boutheina Ben Abdelmoumen Mardassi, Hajer Ayari, Awatef Béjaoui-Khiari, Béhija Mlik, Imed Moalla, Faten Amouna

**Affiliations:** 1 Laboratoire des Mycoplasmes, Institut Pasteur de Tunis, 13, Place Pasteur-B.P.74, 1002 Tunis-Belvédère, Tunis, Tunisie

## Abstract

**Background:**

Among the surface antigens of *Mycoplasma hominis*, the P120' protein was previously shown to elicit a subtle antibody response and appears to be relatively conserved. To get better insight into the evolution of this protein, we analysed the genetic variability of its surface exposed region in 27 *M. hominis *isolates recovered from the genital tract of Tunisian patients with infertility disorders.

**Methods:**

All specimens were processed for culture and PCR amplification of the N-terminal surface exposed region of p120' gene. PCR products were sequenced to evaluate the genetic variability, to test for adaptive selection, and to infer the phylogenetic relationship of the *M. hominis *isolates.

**Results:**

Sequence analysis showed a total of 25 single nucleotide polymorphisms distributed through 23 polymorphic sites, yielding 13 haplotypes. All but one mutation were confined within three distinct regions. Analysis of the amino acid-based phylogenetic tree showed a predominant group of 17 closely related isolates while the remaining appear to have significantly diverged.

**Conclusion:**

By analysing a larger sample of *M. hominis *recovered from patients with urogenital infections, we show here that the P120' protein undergoes substantial level of genetic variability at its surface exposed region.

## Background

*Mycoplasma hominis *belongs to the class *Mollicutes*, the smallest cell-wall free prokaryotic organisms. Mycoplasmas are considered to be a *clostridial *branch of Gram positive *eubacteria *and were supposed to have lost a large part of genomic material and metabolic pathways during their evolution [1,2]. Recently, the *M. hominis *genome size was reported and shown to be composed of 665,445 kpb with 27 mol% of G+C [3]. *M. hominis *is an opportunistic pathogen causing gynaecological infections [4]. It has been associated with pyelonephritis, pelvic inflammatory disease, and postpartum septicaemia [5]. *M. hominis *is increasingly detected in extragenital infections [6], infants born to infected mothers became infected with these bacteria [7] and colonization of the respiratory tract of infants has been associated with pneumonia and meningitis [8,9].

Like the majority of mycoplasma species, *M. hominis *appears to be equipped with a genetic system that allows it *in vivo *to alter its surface exposed, membrane-associated, antigenic repertoire. Three surface membrane proteins, P120, Lmp, and Vaa, whose products undergo genetic variability could account for the ability of this microorganism to circumvent the host immune system [10-13]. The highly antigenic P120 gene displays a hypervariable region due to accumulation of mutations, while Lmp1 and Lmp2 genes show size variations and could be expressed as a chimearic protein. The Vaa gene product, which is involved in cell adherence, displays both size variation and frameshift mutation to create variant products. However, an additional surface protein, related to the variable P120 was identified and named P120' [10]. In contrast to the former, P120' appears to be particularly conserved and weakly recognised by sera from patients with *M. hominis *confirmed infection. It has been speculated that P120 and P120' could have evolved by gene duplication followed by sequence divergence, whose extreme counterpart example is represented by the pMGA gene family of the avian *Mycoplasma gallisepticum *[14,15].

In the present study, we show that the N-terminal surface exposed region of the P120' protein undergoes substantial genetic variability within strains isolated from patients displaying urogenital and infertility disorders.

## Methods

### Mycoplasma strains and growth media

Five reference strains of human mycoplasmas, three ureaplasma isolates, and 27 mycoplasma clinical isolates were used: Reference strains were purchased from the American Type Culture Collection (ATCC): *M. hominis *PG21 (ATCC 23114), *M. fermentans *(ATCC 19989), *M. genitalium *(ATCC 33530), *M. pneumoniae *(ATCC 15531), and *Ureaplasma urealyticum*, isolate UuipT3, recovered in our laboratory. Reference and isolated mycoplasma strains were cultivated in SP-4 medium [16] supplemented with 5% of CMRL 1066 (Sigma), 2000 units/ml of penicillin G, 500 units/ml of polymyxin B, 2.5 μg/ml amphotericin B, 10% of fresh yeast extract (Amersham Biosciences), 15% of horse serum (Gibco BRL), and 0.5% phenol red. The medium was further supplemented with 0.5% of glucose, 0.5% of arginine, or 0.5% of urea depending on the nutritional needs of the species being cultivated. Broth culture was done at 37°C and mycoplasma growth confirmation was performed onto SP-4 agar plates maintained at 37°C with 5% CO_2 _and regularly observed microscopically for the appearance of mycoplasma colonies.

### Clinical specimens

Cervical swabs from 34 female patients suffering from sexually transmissible disease, 23 vaginal specimens from women presenting a number of complications associated to pregnancy, including chorioamnionitis and preterm birth, 29 urethral specimens obtained from men with urethritis, and 6 semen samples from clinical cases associated with infertility. All these specimens were tested for the presence of genital mycoplasmas (*M. hominis*, *M. genitalium*, *M. fermentans*, and *Ureaplasma urealyticum*) by broth and solid culture, then by PCR. The collected specimens were inoculated onto 1.8 ml SP-4 broth medium and then transferred to the laboratory at 4°C within 24 h. Semen samples were also placed in a tube with 1.8 ml SP-4 medium for cultivation attempts. The specimens were processed on arrival or after storage at -80°C. After filtration through 0.45 μm single use syringe filter, 200 μl of the specimen was inoculated into each of SP-4 broth with 0.5% of glucose and 0.5% of arginine for the isolation and identification of mycoplasmas and SP-4U broth with 0.5% of urea for *Ureaplasma urealyticum *isolation and identification. In parallel, 100 μl of the filtrated specimen was inoculated onto each of SP-4 and SP-4U agar plate for the monitoring of mycoplasma or ureaplasma colonies appearance. All broth cultures were incubated at 37°C and examined daily for turbidity and pH change. The broth cultures were subcultured onto agar plates once their pH changes. The plates were incubated at 37°C in a 5% carbon dioxide environment and examined every two days for the appearance of mycoplasmas or ureaplasma colonies. To confirm the identification of Mycoplasma spp or ureaplasma, PCR assays have been used on positive broth cultures. For mycoplasma titration, dilution culture and subculture were achieved as described elsewhere [17].

This study has been conducted in close conformity with ethical aspects, which were established by the ethical committee of the Tunisian Ministry of Health. The samples were collected in the context of the routine diagnostic activity of the laboratory of Mycoplasma and with the consent of the patients. All the patient files are confidential and none could be identified.

### DNA extraction and sample preparation for PCR amplification

Culture (8 ml) of the cloned mycoplasma strains and clinical samples was centrifuged at 17 000 rpm for 30 min, washed in PBS (0.1 M-NaCl, 2.5 mM-KCl, 10 mM-Na_2_HPO_4_, 1.5 mM-KH_2_PO_4_, pH 7.4), and then suspended in 200 μl of PBS. This concentrated culture was processed for PCR as described elsewhere [18]. Briefly, SDS to a final concentration of 1% was added to the DNA and incubated at 37°C for half an hour in the presence of RNase A (5 μg/ml). A volume of 25 μl was then used for PCR amplification.

### Primers and PCR amplification

Two pairs of primers were used in this study. The pair Mhp120'F (5'-GAGGAATTTCAACTGGTGTCC-3') and Mhp120'R (5'-CTGTTGTAATAGCATTTAAG-3') was specific to the *M. hominis *p120' gene. PCR was performed on 25 μl of treated sample in a total volume of 50 μl containing, 10 mM Tris-HCl (pH 8.3), 50 mM KCl, 2.5 mM MgCl_2_, 250 μM of each dNTP (Amersham Biosciences), 50 pmol of each primers, and 1.25 U of *Taq *DNA polymerase (Amersham Biosciences). The PCR mixtures were subjected to 30 cycles consisting of 1 min at 95°C, 2 min at 50°C, and 1 min at 72°C in a Perkin-Elmer GeneAmp PCR System 9700 thermocycler. After the last cycle, a final extension temperature of 72°C was maintained for 10 min. To determine the specificity of *M. hominis *primers, the PCR reaction was performed with DNAs from other mycoplasma species, *M. fermentans*, *M. genitalium*, *M. pneumoniae*, which occasionally has been isolated from urogenital tract, and *Ureaplasma urealyticum*, strain UuipT3. The PCR products were analysed on 2% agarose gels. The PCR experiments were repeated at least twice for results confirmation.

### Sequence analysis

The PCR products were separated by electrophoresis in a 1.5% low-melting-point agarose gel, excised from the gel, and then purified using the GFX PCR DNA and Gel Band Purification system (Amersham Biosciences). Determination of the nucleotide sequence was performed with the Prism Ready Reaction Dye Deoxy Terminator Cycle sequencing Kit on an ABI PRISM 377 DNA sequencer (Applied Biosystems). Each sample was sequenced from two independent PCR amplification reactions.

The sequence data (GenBank accession numbers; pending) were aligned using ClustalW [19] and edited with the software programs BioEdit [20]. DNASP [21] was used to calculate haplotypes and to test for adaptive selection, by determining the nucleotide substitution changes and the ratio of synonymous (Ks) and nonsynonymous (Ka) substitutions per site. For this purpose, we used the analysis developed by Nei-Gojobori [22] as implemented in the DNASP package. A dendrogram illustrating the genetic relatedness of the recovered *M. hominis *isolates was constructed using MEGA version 3.0. [23]. The dendrogram was obtained using the neighbor-joining method with 1000 bootstrapping replicates.

## Results

### Isolation, titration and cloning of *M. hominis *strains

To determine the initial count of the mycoplasma species contained within the clinical samples, ten-fold dilutions of the original sample were performed and inoculated to SP-4 agar plates. Among the clinical specimens, 27 yielded a high number of viable mycoplasma varying from 10^5 ^to 10^6 ^C.F.U. (colony forming unit)/ml for *M. hominis *and only 5 *M. genitalium *and 12 *U. urealyticum *isolates were detected by culture with low number (≤ 10^4 ^C.F.U/ml) (Tab. 1). As mycoplasma cultures obtained from clinical specimens often represent a mixture of several species, especially with *M. hominis *and *U. urealyticum*, cloning was undertaken so that PCR amplification would involve a DNA template prepared from a single clone. Cloning is also crucial to obtain a real picture of the genetic heterogeneity within a single specie. For this purpose, single colonies from the highest (often the fifth or the sixth dilution) ten-fold dilutions of each specimen culture were picked, cultured into SP-4 broth medium, then diluted through 10 serial dilutions, and finally, 100 μl of each dilution was subcultured onto a SP-4 agar plate. This process was repeated thrice and allowed the appearance of typical *Mycoplasma hominis *colonies in these 27 high tittering clinical specimens. The colonies are clearly distinguishable from those of *U. urealyticum *which are characterized by their tiny size and dark colour.

### PCR amplification and genetic diversity of the P120' surface exposed N-terminal coding sequence

**Figure 1:**
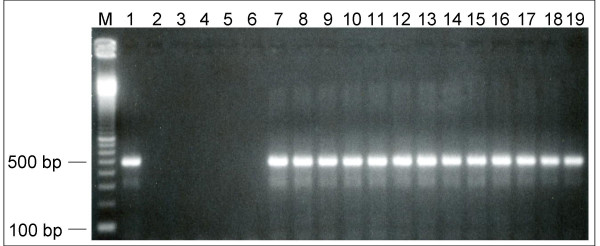
Representative illustration of PCR amplification of the 510-bp fragment of *M. hominis*. M: 100 bp molecular size ladder, lanes 1 and 7: *M. hominis *PG21 strain (positive control), lane 2: No *M. hominis *DNA with Mhp120' primers (negative control), lanes 3–6: Mhp120' primers with DNA from *M. genitalium *ATCC 33530 (lane 3), *M. fermentans *ATCC 19989 (lane 4), *M. pneumoniae *ATCC 15531 (lane 5), and *U. urealyticum *UuipT3 (lane 6), lanes 8–19: *M. hominis *isolates.

All the 27 *M. hominis *cloned isolates yielded a PCR product of the expected molecular size (510 bp), suggesting that this gene sequence is highly conserved (Fig. 1). No amplification was observed using DNAs from other mycoplasma species namely, *M. genitalium*, *M. fermentans*, *M. pneumoniae *and *U. urealyticum *(Fig. 1, lanes 3–6), thus confirming the specificity of the PCR reaction. The nucleotide sequence encompassing the positions 145 to 565 (421 nucleotides) of the P120' gene sequence (amino acids 49 to 188) was obtained for each sample, by sequencing in both directions, two PCR products from independent amplifications. A total of 25 single nucleotide polymorphisms (SNPs) distributed through 23 polymorphic sites were observed (A162G, T177A, T192G, A212T, A212G, A215G, C234T, T297C, A300G, A300C, A379C, G394A, C396T, A399T, C405T, A409T, A417T, T441A, A482T, C483T, G500T, C509T, A552G, A563G, and T564A), yielding 13 haplotypes. Mutations at positions 234 (C to T) and 394 (G to T) were shared by the 27 isolates, whereas the SNPs at positions 297, 300, 379, 396, 405, 500, and 552 were found in more than 20 isolates.

**Figure 2:**
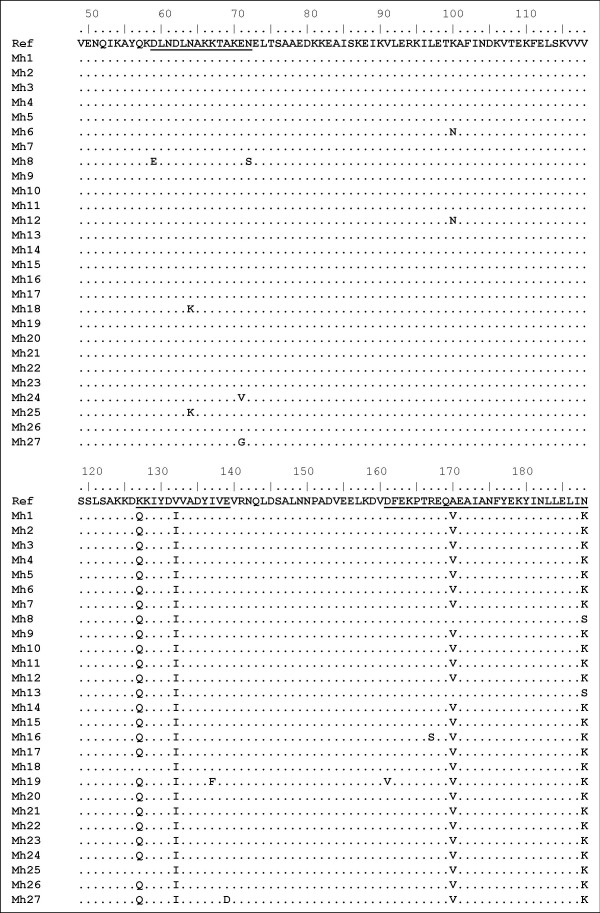
Multiple alignment of the deduced amino acid sequences of the P120'surface exposed region of *M. hominis *isolates (Mh1 to Mh27) relative to the reference strain PG21. Dots indicate identical residues. The 3 regions where amino acid changes which tend to occur are underlined.

Among the 23 polymorphic sites, thirteen (56.5%) are non synonymous as they cause an amino acid change (D59E, N64K, E71V, E71G, N72S, K100N, K127Q, V132I, I137F, E139D, D161V, R167I, A170V, and N188R) (Fig. 2). Of these, mutations K127Q, V132I, A170V, and N188K were shared by most of the isolates. Overall, the mutations were mainly confined within three regions, 59–72, 127–139, and 161–188, each containing four amino acid changes. We could distinguish 10 haplotypes among the deduced amino acid sequences.

### Genetic relatedness of *M. hominis *isolates and evolution of the N-terminal region of P120'

**Figure 3:**
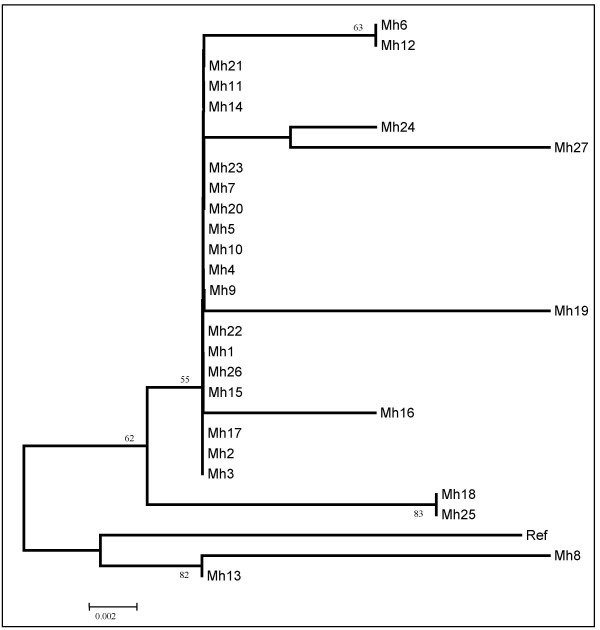
Dendrogram showing the phylogenetic relationships among the 27 *M. hominis *isolates based on their deduced amino acid sequences. The neighbour-joining-based consensus tree was generated upon 1000 data sets. Ref: *M. hominis *PG21 strain.

The unrooted neighbour-joining-based phylogenetic derived tree (Fig. 3), using the deduced amino acid sequences of the 27 *M. hominis *isolates, showed a major branch containing a group of 17 closely-related isolates and 6 variant emerging strains. This major branch, which is supported by bootstrap, is clearly distinct from that of the reference *M. hominis *strain PG21. The remaining isolates are less phylogentically close to the majority of the pool; Mh13 and Mh8 belonging to a distinct evolutionary tract.

The acquisition rates for sSNP (Ks) and nsSNP (Ka) were calculated in order to evaluate the evolutionary trend of this surface exposed part of the P120' protein. We found that the overall mean value of 0.046 for Ks (0.011–0.47) is significantly different (*P *< 0.0001) from the 0.012 mean value of Ka (0.003–0.18). The ratio Ka/Ks (0.26) is thus <1, indicative of a negative selection. This finding is in accordance with a previous study involving house-keeping genes [24].

## Discussion

*M. hominis *is commonly associated with the normal flora of the female genital tract, in many instances, it was also isolated at high titers from patients with clinical manifestations [7]. Factors relating to host (impairment of local immunity) and to *M. hominis *(essentially selection of immune escape mutants and perturbation of the host cell) have been suggested as a basis for the disruption of the host-pathogen equilibrium and the appearance of clinical disorders [25].

Because *M. hominis *in the processed samples was at significant high titers, we reasoned that sufficient replication would have occurred in the face of the immune system of the host and, thus, it could be an opportunity to evaluate the genetic variability of surface exposed proteins. We focused our analysis on P120' protein, a homolog of P120, which unlike the latter, seems to be less subjected to genetic variability [10,26], and whose function remains to be determined. We targeted the N-terminal region of the P120' protein as it has been previously shown to be surface exposed [10]. Our results show that significant variability among the different isolates has accumulated, although the time frame in which the isolates were obtained is relatively short. This finding is in fair agreement with a previous study that showed that *M. hominis *isolates from different individuals always differed, a finding explained by the occurrence of intergenic recombination [24]. One can argue that higher variability would have been observed if samples collected over extended time periods were analysed. However, one cannot also dismiss the possibility that increased genetic variability could have been observed among isolates belonging to different geographical origins. This might be reflected, in our study, by the fact that the English *M. hominis *reference strain, PG21, differed from most of the Tunisian isolates in 4 of the varying sites. In fact, the latter strain also differed from all the isolates included in Sogaard's study. As far as could ascertained, as a reference strain, PG21 could have been subjected to several passages and transferred to many laboratories, thus contributing to its apparent divergence.

All but one mutation was confined within three distinct regions, suggesting that they might be under selective pressure. Hence, although the surface exposed region, analysed as a whole, appears to be naturally under negative selection (Ka/Ks < 1), the three main variable regions might evolve towards the accumulation of higher non synonymous changes, and may thus represent variable evolving domains. Within the pool of recovered isolates, a predominant group of closely-related strains was identified. Phylogenetic analysis showed that variants from this group tend to emerge. It remains to be seen, through analysis of larger samples over extended periods of time, whether emergence of these variants is a result of the host immune pressure. It is worthy of mentioning that P120' is weakly recognised by patient sera, thus immune selection may occurs through long-term periods or it may be the result of a cellular immune response.

## Conclusion

Overall, this study showed that the homolog of the variable surface exposed P120 protein, P120', was subjected to genetic variability in its surface exposed domain despite its low reactivity with patient sera. Since both genes seem to have evolved through gene duplication and genetic divergence, they would certainly assume distinct essential functions as they are both conserved and expressed within a single strain. This situation contrasts with other mycoplasmas, such as the avian *M. gallisepticum *[15] and *M. synoviae *[27], whereby only one member of the family of the variable antigens is expressed. *M. homonis *does not seem to follow this restrictive strategy as, apart from P120 and P120', at least two other variable proteins, Lmp and Vaa, contribute to its variability.

## List of abbreviations

SNP: Single nucleotide polymorphism

sSNP: synonymous single nucleotide polymorphism

nsSNP: nonsynonymous single nucleotide polymorphism

## Competing interests

The author(s) declare that they have no competing interests.

## Authors' contributions

All authors read and approved the final manuscript. BBAM, overviewed the work, performed the sequencing analysis and wrote the manuscript. HA, conducted the PCR amplification and performed the sequencing reactions. ABK, repeated and confirmed the sequencing analysis and helped in reviewing the manuscript. BM and FA performed the clinical specimens' treatment, the mycoplasmas culture and cloning. IM, carried out the PCR amplification of *Mycoplasma hominis *isolates and other genital Mycoplasma spp for specificity evaluation.

## Pre-publication history

The pre-publication history for this paper can be accessed here:


